# From support to homeostatic licensing: the bidirectional fibroblast–macrophage circuit and its central role in tissue homeostasis and fibrosis

**DOI:** 10.3389/fimmu.2026.1917469

**Published:** 2026-07-22

**Authors:** Meng Zhang, Ruiqi Chu, Shengni Zhang, Xiangxiang Ren

**Affiliations:** 1 Department of Dermatology, Affiliated Hospital of Hebei University, Baoding, Hebei, China; 2 Department of General Surgery, Affiliated Hospital of Hebei University, Baoding, Hebei, China

**Keywords:** bidirectional regulation, cell niche, CSF1–CSF1R axis, fibroblasts, fibrosis, homeostatic licensing, macrophages, tissue homeostasis

## Abstract

The capacity of fibroblasts to support macrophages predominantly through colony-stimulating factor 1 (CSF1)-mediated survival and proliferation has long been recognized. Whether macrophages actively regulate fibroblasts *in vivo* has remained a critical open question. Recent work in murine skin has provided the first direct evidence for reverse homeostatic licensing, a process whereby macrophages, in return, provide permissive signals that maintain fibroblast quiescence: conditional deletion of Csf1 in dermatopontin-expressing (Dpt+) fibroblasts causes a progressive loss of CD64+ and CD11c+ macrophages, which in turn deprives fibroblasts of essential microenvironmental cues, leading to disrupted cell-cycle, metabolic, and immune signaling programs, and compensatory fibroblast expansion. This discovery formally defines a bidirectional circuit. In human systemic sclerosis, elevated fibroblast-derived CSF1 and increased macrophage abundance jointly correlate with disease severity, a paradox we resolve through the concept of functional licensing exhaustion in disease-associated macrophages. Taking this breakthrough as a point of departure, we integrate the concept of a fibroblast-macrophage homeostatic circuit as a generalizable framework. We examine how this push-pull loop operates in tissue maintenance, wound repair, and fibrogenesis; explore its similarities and divergences in the tumor microenvironment and across organ fibroses; and propose a therapeutic shift from simple cell depletion to the restoration of circuit equilibrium. The framework provides a conceptual basis for clinical strategies that target stromal-immune interactions.

## 1 Introduction: from unidirectional support to bidirectional crosstalk

A recent study by Vollmers et al. (1), using genetic mouse models, single-cell multi-omics, flow cytometry, and imaging, has transformed this landscape. The authors showed that either depletion of dermal fibroblasts or the selective loss of fibroblast-derived growth factors profoundly alters macrophage populations in the dermis and subcutis. Crucially, when macrophage numbers declined following Csf1 deletion in dermatopontin-expressing (Dpt+) dermal fibroblasts, a subset distributed across the dermis and adventitial regions that serves as a key resource-cell population for skin macrophages, the fibroblasts themselves exhibited transcriptomic disturbances characterized by dysregulation of cell cycle control, metabolism, and immune signaling programs, consistent with a de-repression type of fibroblast activation. This was accompanied by a compensatory increase in fibroblast number. Importantly, the authors observed convergent fibroblast phenotypes when macrophages were directly depleted, arguing against a purely cell-autonomous effect of CSF1 withdrawal on fibroblasts and supporting a macrophage-dependent relay mechanism. We note, however, that formal proof of this relay, for example, through macrophage adoptive transfer into Csf1-deleted backgrounds, remains an important direction for future studies (1).

### 1.2 A conceptual leap to bidirectionality

A recent study by Vollmers et al. (1) has transformed this landscape. Using genetic mouse models, single-cell multi-omics, flow cytometry, and imaging, the authors showed that either depletion of fibroblasts or the selective loss of fibroblast-derived growth factors profoundly alters macrophage populations in the dermis and subcutis. Crucially, when macrophage numbers declined following Csf1 deletion in Dpt^+^ fibroblasts, the fibroblasts themselves exhibited striking disturbances in cell cycle control, metabolism, and immune signaling, accompanied by a compensatory increase in their own number. This provides the first direct *in vivo* demonstration that macrophages actively regulate fibroblast number and function, and it exposes a genuine bidirectional communication network.

We advance the following proposition: macrophages are not passive recipients of fibroblast-derived growth factors, but active regulators of fibroblast state. To avoid ambiguity, the term constraint is not meant to imply direct killing or suppression. Instead, we use homeostatic licensing to describe the provision by macrophages of an essential, permissive cue that keeps fibroblasts quiescent. Loss of macrophages removes this cue, leading to a de-repression type of fibroblast activation.

It is useful at the outset to distinguish three categories of intercellular signals that often become conflated in the literature. Trophic support encompasses factors such as CSF1 and IL-34 that promote survival, proliferation, or differentiation of the recipient cell, the classic fibroblast-to-macrophage forward signal. Homeostatic licensing, by contrast, describes a permissive signal that does not necessarily drive proliferation or differentiation but is required to maintain the target cell in a quiescent, baseline state; its withdrawal triggers latent activation programs. Lastly, direct inhibitory signals would actively repress proliferation or synthetic functions. The work discussed below strongly favors a licensing mechanism, because the fibroblast response to macrophage loss unfolds as a progressive, YAP/TAZ-dependent de-repression of cell-cycle and metabolic programs. YAP (Yes-associated protein) and TAZ (transcriptional coactivator with PDZ-binding motif) are Hippo pathway transcriptional coactivators that function as mechanosensitive relays promoting proliferation, survival, and matrix synthesis. The progressive nature of de-repression argues against an immediate, unleashed burst of proliferation that would be expected upon removal of a direct brake. Keeping these distinctions in mind clarifies the circuit logic throughout the review.

At this juncture, a clear contradiction must be acknowledged. In fibrotic diseases such as systemic sclerosis, both CSF1 levels and macrophage numbers are elevated (2), not reduced. This appears to oppose the idea that macrophage loss triggers fibroblast activation. We resolve this paradox later by introducing the concept of “functional licensing exhaustion, “ whereby disease-associated macrophages, despite their abundance, lose the capacity to deliver homeostatic cues and may even become actively pro-fibrotic. Imbalance in the bidirectional circuit—whether arising from defective fibroblast CSF1 secretion or from a qualitative failure of macrophage licensing function—is a key driver of tissue homeostasis collapse and fibrosis (3). Using the recent *in vivo* evidence from skin (1) as an empirical anchor, we extend this circuit model to scleroderma, the tumor microenvironment, and multi-organ fibrosis, and discuss its profound implications for therapeutic design.

Finally, it should be recognized that CSF1 is not an exclusive product of fibroblasts. Vascular endothelial cells, epithelial cells, osteoblasts, and subsets of lymphocytes and myeloid cells also secrete CSF1, and the relative contribution of each source likely varies by tissue, developmental stage, and disease context (4, 5). This cellular redundancy in CSF1 production adds a layer of complexity to the circuit: macrophage homeostasis may be partially buffered when fibroblast-derived CSF1 is compromised, yet the qualitative licensing signals provided by macrophages may still depend on the specific fibroblast subset that serves as their niche. Moreover, the concept of bidirectional homeostatic regulation is unlikely to be limited to the fibroblast-macrophage pair. Analogous reciprocal relationships may operate between pericytes and microglia, osteoblasts and osteoclasts, or fibroblasts and mast cells, circuits that remain to be formally identified and characterized.

## 2 Bidirectional regulatory mechanisms in the skin

### 2.1 The forward push: fibroblast niche support for macrophages

Fibroblasts are heterogeneous. The dermatopontin-expressing (Dpt+) subset, introduced above, has been identified as a key resource-cell population distributed across the dermis and adventitial regions. In murine genetic models, selective ablation of Dpt+ fibroblasts markedly reduced multiple transcriptional states of dermal fibroblasts and concurrently decreased total CD11b+F4/80+ macrophages, affecting both CD64+ and CD11c+ subpopulations (1). These results establish Dpt+ fibroblasts as a critical interstitial niche for skin macrophages.

Within this niche, CSF1 is the dominant molecular bridge. Conditional deletion of Csf1 specifically in Dpt+ fibroblasts in mice caused a progressive decline in CD64+ and CD11c+ macrophages, whereas deletion of the alternative CSF1R ligand IL-34 had minimal impact (1). This pattern echoes observations in the intestinal mucosa, where fibroblast-derived CSF1 is the principal factor maintaining mucosal macrophage colonization, with spatial compartmentalization of CSF1 and IL-34 sources (5). It remains to be determined whether an analogous spatial organization of niche signals exists in skin.

Skin macrophages comprise CD64+ tissue-resident cells with self-renewal capacity and CD11c+ monocyte-derived populations (1, 6–10). In the murine genetic model, Csf1 deletion in Dpt+ fibroblasts led to a reduction in both subsets, albeit with differences in kinetics, pointing to differential reliance on fibroblast-derived CSF1 (11). The distinct kinetics likely reflect the higher CSF1R expression and local proliferative capacity of resident macrophages compared with their monocyte-derived counterparts (12). Single-cell analysis additionally revealed a relative expansion of Spp1+ (osteopontin-positive) inflammatory myeloid cells as the absolute numbers of homeostatic macrophages declined (13, 14). This compositional shift marks a transition of the immune microenvironment away from homeostatic maintenance and toward an inflammatory bias.

### 2.2 The reverse pull: homeostatic licensing of fibroblasts by macrophages

The most consequential finding is the reverse regulation of fibroblasts by macrophages. When macrophage abundance fell because of CSF1 deficiency in the murine skin, fibroblasts underwent deep transcriptomic reprogramming involving cell cycle control, anabolic metabolism, and immune signaling. Mechanistically, the YAP/TAZ signaling axis, comprising the Hippo pathway transcriptional coactivators YAP (Yes-associated protein) and TAZ (transcriptional coactivator with PDZ-binding motif), which function as mechanosensitive relays that promote proliferation, survival, and matrix synthesis, was de-repressed, a pathway already implicated in fibrosis in multiple tissues (1, 15). Receptors such as Axl may also participate in sensing the absence of macrophages. We note that formal proof of the macrophage-dependent relay mechanism, for example, through macrophage adoptive transfer experiments into Csf1-deleted backgrounds, remains an important direction for future studies.

A critical interpretive issue arises, however. The concurrent relative expansion of Spp1^+^ inflammatory myeloid cells could actively deliver pro-fibrotic stimuli, such as osteopontin, rather than the fibroblast activation being solely the result of withdrawn licensing cues (16). The two scenarios are not mutually exclusive and likely act cooperatively: the loss of quiescence-maintaining signals is reinforced by newly emerging pro-inflammatory and pro-fibrotic mediators. Disentangling their relative contributions will require experiments that specifically neutralize Spp1^+^-derived signals (17)—for example, by antibody blockade or conditional deletion of Spp1 (18)—in the context of macrophage depletion, as well as the direct identification of the macrophage-derived molecule(s) that enforce fibroblast quiescence (19). For the present, it is most accurate to view macrophages as providers of a permissive, multi-factorial license, the withdrawal of which unleashes latent pro-proliferative and pro-synthetic programs. At the same time, it must be acknowledged that a component of the observed fibroblast activation is likely driven actively by the signals emerging from the altered myeloid compartment (17), underscoring the circuit’s dual susceptibility to loss of tonic signals and gain of pathologic ones (20).

As to the molecular nature of the licensing cue itself, the answer remains unknown. Several plausible candidates have been proposed based on the pathways known to counteract YAP/TAZ activity and maintain stromal quiescence. Secreted Wnt antagonists, such as the secreted Frizzled-related proteins and Dickkopf family members, can dampen proliferative beta-catenin signaling, the Wnt/beta-catenin pathway is a conserved signaling cascade governing cell proliferation and fate decisions, and are expressed by certain macrophage populations (21, 22). Bone morphogenetic proteins (BMPs), which in other contexts preserve a quiescent mesenchymal phenotype, represent another class of candidate licensing factors (23, 24). Additionally, the Gas6 (growth arrest-specific 6)/Pros1 (protein S)-Axl receptor tyrosine kinase axis, which transmits anti-inflammatory and pro-efferocytic signals and can suppress YAP/TAZ nuclear translocation, is worth investigating: Axl expression is reduced when macrophages are lost (1). That said, none of these candidate molecules have been shown *in vivo* to be macrophage-derived signals that keep fibroblasts quiescent. Identifying the complete set of signaling molecules underlying macrophage licensing remains a major goal in the field.

The transcriptomic rewiring translated directly into a quantitative phenotype: macrophage decline was followed by a compensatory increase in fibroblast number and a measurable thickening of the dermis. This inverse relationship is the most direct *in vivo* evidence that macrophages control fibroblast abundance (1). Notably, an earlier study performed in mice using a Col1a2-Cre driver to delete Csf1 reduced subcutaneous macrophages without significantly changing fibroblast gene expression, suggesting that distinct fibroblast subsets or anatomical compartments differ in their responses to macrophage feedback (25).

#### 2.2.1 Dual-end disruption of the CSF1–CSF1R axis

The integrity of the CSF1–CSF1R axis is required to maintain bidirectional equilibrium (25, 26). When Csf1 is deleted from fibroblasts, both the forward nurturing function and the reverse licensing mechanism collapse (1, 25). This raises a clinically important distinction: pharmacological CSF1R blockade might not only deplete macrophages but also leave behind a population of numerically normal yet functionally incapacitated cells—macrophages that fail to license fibroblasts and may adopt a pathological phenotype. Whether a therapeutic strategy should aim to restore, reprogram, or selectively eliminate such dysfunctional macrophages remains an open and crucial question.

## 3 Pathological consequences of circuit imbalance

In a murine skin excision wound-healing model, conditional deletion of Csf1 in Dpt+ fibroblasts reduced macrophage numbers and markedly delayed wound closure. The defect was directly attributed to a deficiency in CD206+ reparative macrophages, cells that normally supply factors for ordered fibroblast proliferation and clearance of debris. Viewed through the circuit lens, failed wound repair illustrates that the bidirectional loop is not a static fixture but a dynamic system that must be transiently boosted upon injury. When CSF1 supply is interrupted, this adaptive engagement stalls from the outset (1).

Loss of macrophage licensing also drives fibrotic predisposition. In the murine model, macrophage decline was accompanied by compensatory fibroblast accumulation and deposition of extracellular matrix components such as hyaluronic acid (HA) in the dermal tissue (1). Increased HA deposition raises tissue stiffness and can further promote sustained fibroblast activation via integrin signaling and mechanotransduction (1, 27), setting up a vicious cycle. The expanded Spp1+ myeloid population may additionally contribute pro-fibrotic stimuli (17, 28, 29). It is probable that both the withdrawal of quiescence-maintaining licensing cues and the active delivery of pro-fibrotic signals by emergent myeloid cells conspire to convert fibroblasts from homeostatic custodians into pathological effector cells (30). Definitive proof of this cooperative model awaits lineage-specific perturbation studies that can isolate the contribution of each arm.

**Figure 1:**
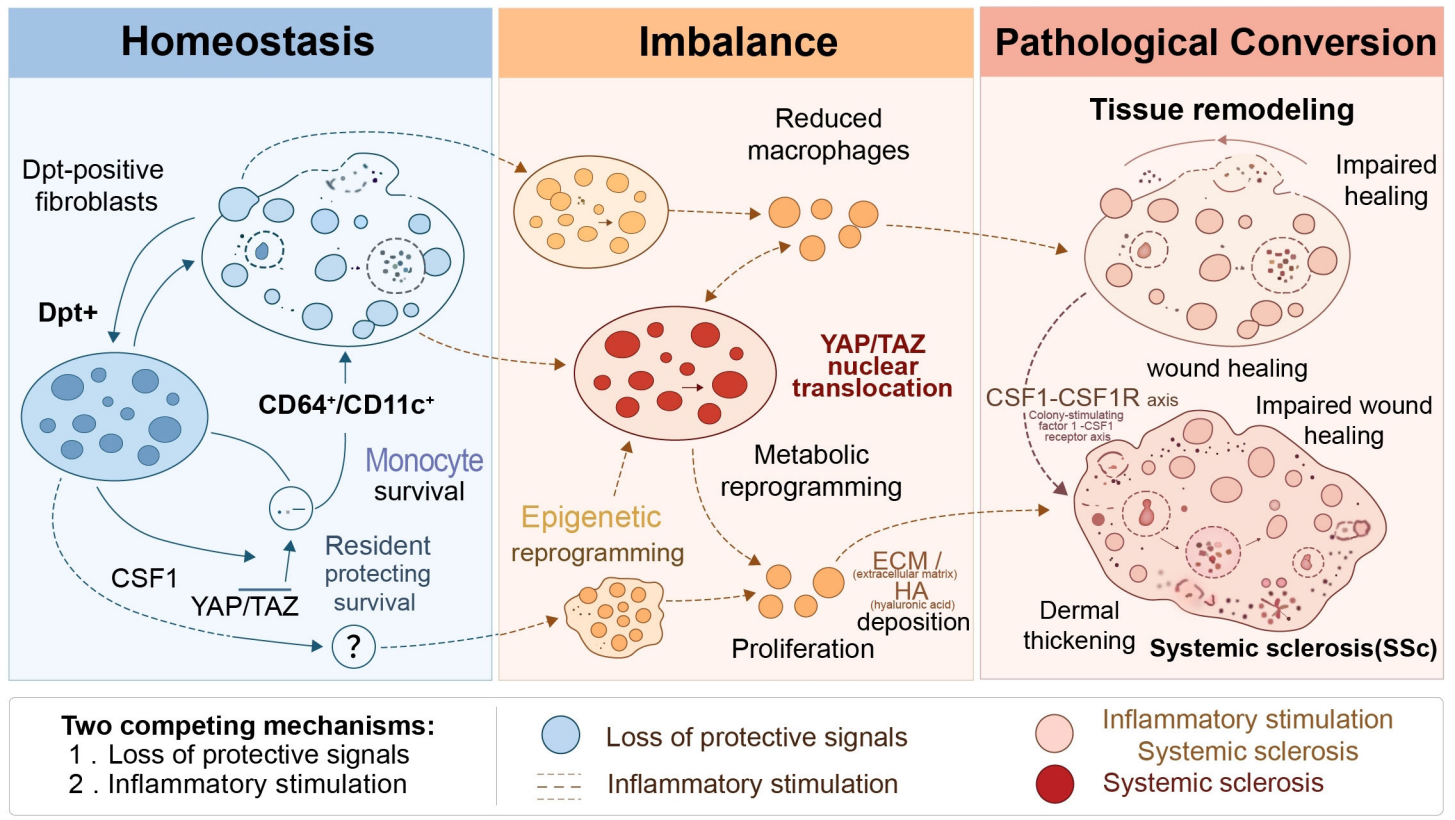
Bidirectional fibroblast–macrophage signaling circuit governing tissue homeostasis, homeostatic imbalance, and pathological conversion toward systemic sclerosis. (Left panel, Homeostasis): Dpt⁺ fibroblasts secrete CSF1 (blue arrows), which sustains the CD64⁺CD11c⁺ resident macrophage pool via CSF1R signaling. In turn, macrophages deliver homeostatic inhibitory cues (candidate mediators including Wnt antagonists, BMPs, and Gas6/Pros1) that restrict YAP/TAZ nuclear translocation and maintain fibroblast quiescence, establishing balanced fibroblast and macrophage populations. (Middle panel, Imbalance): Disruption of the CSF1–CSF1R axis (e.g., fibroblast-specific Csf1 ablation) results in reduced macrophage abundance. Loss of macrophage-derived protective licensing signals relieves repression of YAP/TAZ, enabling YAP/TAZ nuclear translocation to drive fibroblast proliferation, epigenetic and metabolic reprogramming, and enhanced extracellular matrix production. Concurrently, pro-fibrotic myeloid populations expand, triggering compensatory fibroblast accumulation and the initial onset of dermal thickening. (Right panel, Pathological Conversion): Under physiological conditions, tissue injury transiently amplifies this circuit to support ordered wound healing. In systemic sclerosis (SSc), chronic circuit dysregulation drives pathological tissue remodeling with impaired wound healing and progressive dermal thickening. Persistently activated fibroblasts hypersecrete CSF1 to recruit macrophages; these disease-associated macrophages lose the capacity to supply fibroblast-quiescing signals and adopt pro-fibrotic phenotypes (secreting TGF-β, PDGF and osteopontin). Sustained YAP/TAZ nuclear activity in fibroblasts, combined with the self-perpetuating CSF1–CSF1R signaling loop, fuels unremitting matrix deposition and progressive tissue fibrosis. Abbreviations: Dpt, dermatopontin; CSF1, colony-stimulating factor 1; CSF1R, CSF1 receptor; YAP, Yes-associated protein; TAZ, transcriptional coactivator with PDZ-binding motif; Spp1, secreted phosphoprotein 1 (osteopontin); BMPs, bone morphogenetic proteins; Gas6, growth arrest-specific 6; Pros1, protein S; TGF-β, transforming growth factor-β; PDGF, platelet-derived growth factor.

In systemic sclerosis, elevated fibroblast-derived CSF1 and increased macrophage infiltration correlate with disease severity in patient skin biopsy analyses (1). To reconcile this with the inverse relationship seen in genetic mouse models, we propose a staged concept of functional licensing exhaustion. In early disease, intrinsic fibroblast activation drives increased CSF1 secretion, actively recruiting macrophages. Yet, in the chronic fibrotic milieu, recruited macrophages undergo a fundamental functional shift. Single-cell atlases of fibrotic tissues across organs have repeatedly identified macrophage subsets, commonly marked by Trem2, Spp1, and a lipid-associated phenotype, that are transcriptionally distinct from homeostatic tissue-resident macrophages and have lost niche-supporting capacity (17, 31–33). These cells may even become actively pro-fibrotic, secreting TGF-beta, PDGF, and osteopontin. The persistently elevated CSF1 thus reflects a futile compensatory attempt: fibroblasts signal for a license that incoming macrophages can no longer grant, perpetuating a vicious cycle. It remains to be determined whether this functional exhaustion represents a stable epigenetic commitment or a reversible state that could be therapeutically restored (1, 34, 35). Spatially resolved transcriptomics will be essential to validate this staged dynamic and to clarify causality in scleroderma lesions. A schematic of this circuit in the context of systemic sclerosis is provided in **Figure 1**.

## 4 Cross-tissue and cross-disease generalizability

The bidirectional circuit is not restricted to skin. However, generalizability does not imply identity; organ-specific variations are pronounced. **Table 1** summarizes the core characteristics, key differences, and speculated licensing signals across organs.

**Table 1:** Common features and key distinctions of the fibroblast–macrophage homeostatic circuit across organs.

Feature	Skin	Liver	Lung	Tumor microenvironment
Core stromal cell	Dpt^+^ fibroblasts (4)	Hepatic stellate cells (HSCs)	Alveolar/interstitial fibroblasts	Cancer-associated fibroblasts (CAFs)
Key macrophage population	CD64^+^/CD11c^+^ tissue-resident macrophages (35)	Kupffer cells/monocyte-derived macrophages (36)	Alveolar/interstitial macrophages (37)	Tumor-associated macrophages (TAMs) (38)
Core “push” (CSF1 dependence)	High; dominated by Dpt^+^ fibroblast-derived CSF1 (4)	Moderate; Kupffer cells have strong self-renewal, partially HSC-independent	High, especially in fibrotic regions	High; sources include tumor cells and CAFs
Primary “licensing” signal (evidence/candidates)	Unknown; YAP/TAZ and Axl de-repressed upon loss; candidates: Gas6/Pros1–Axl, Wnt antagonists, BMPs	Steady-state licensing unclear; candidate morphogens: Wnts; upon injury, Kupffer cell-derived TGF-β and PDGF override licensing	Unknown; YAP/TAZ de-repressed, modulated by mechanical forces; candidates: Wnt inhibitors, BMPs	Licensing function, if any, is lost or overridden; the circuit is rewritten by tumor-derived CSF1 and inflammatory signals
Consequences of circuit imbalance	Delayed wound healing, spontaneous dermal thickening, HA deposition	Liver fibrosis, cirrhosis	Pulmonary fibrosis	Immunosuppression, desmoplasia, therapy resistance
Therapeutic implications	Restore homeostatic signals; reprogram rather than deplete macrophages	Functional reprogramming of macrophages; combination therapy targeting HSC activation	Modulate mechanotransduction; reprogram reparative macrophage phenotypes	Block CAF compensatory pathways; combine CSF1R and CXCR2 targeting
Special modifiers	Microbiome, high-frequency mechanical injury	Portal endotoxin, metabolic stress	Cyclic mechanical stretch (breathing)	Hypoxia, low pH, tumor secretome

The TME contains a cellular communication network that structurally mirrors the normal stromal-immune circuit. Cancer-associated fibroblast (CAF)-tumor-associated macrophage (TAM) crosstalk regulates tumor progression, metastasis, and therapy resistance. However, the polarity is largely rewritten. Tumor cells themselves become a major ectopic source of CSF1, destroying the normal logic of niche feedback (36–38). A study by Kumar et al. provided a telling example: tumor cell-derived CSF1 suppressed granulocyte-specific chemokine expression in CAFs via an HDAC2-mediated mechanism, thereby limiting pro-tumoral granulocyte migration (39). When CSF1R inhibitors were then used to target TAMs, CAFs responded by inducing infiltration of polymorphonuclear myeloid-derived suppressor cells (PMN-MDSCs), neutralizing the anti-tumor effect (39). This phenomenon demonstrates a pathological compensatory network rather than the simple removal of a homeostatic license. There is currently no evidence that a physiological reverse licensing function of TAMs operates in the TME; the CAF response to macrophage loss is more parsimoniously explained by niche compensation in a circuit already radically altered by tumor-derived factors (40). The TME thus serves as a cautionary tale of what occurs when the circuit is hijacked, emphasizing the risk of perturbation without understanding the rewritten control logic. It is worth asking, however, whether certain TAM subsets that retain features of tissue-resident macrophages (41, 42), for example, those expressing folate receptor 2 (FOLR2) or lymphatic vessel endothelial hyaluronan receptor 1 (LYVE1), markers of a tissue-resident macrophage subset with putative niche-supporting functions, might still possess residual licensing capacity that is simply overridden by dominant tumor-derived signals. Testing this possibility could refine macrophage-reprogramming strategies in oncology.

Bidirectional macrophage-fibroblast interactions are broadly operative in organ fibrosis. In the lung, activated macrophages and myofibroblasts form a pro-fibrotic feed-forward loop (43–46), and the YAP/TAZ pathway has been validated as a key regulator of macrophage-driven pulmonary fibrosis (47). The anti-fibrotic drug nintedanib, in part, exerts its effects by inhibiting CSF1R and promoting a tissue-repair macrophage phenotype rather than simply depleting macrophages (48–50). In the liver, hepatic stellate cells (HSCs) engage in bidirectional cross-talk with Kupffer cells and infiltrating macrophages (51). While HSCs support macrophages partly through CSF1, macrophages regulate HSC activation via TGF-beta and PDGF (52–57). In both organs, single-cell studies have identified disease-associated macrophage populations that have shed their homeostatic signature and adopt pro-fibrotic phenotypes, mirroring the functional licensing exhaustion we propose for skin (17, 31–33). These cross-tissue parallels underscore a unifying circuit logic: when macrophages lose their licensing capacity, fibroblasts default to a pro-fibrotic state regardless of the initiating organ-specific insult.

## 5 Therapeutic implications and future directions

### 5.1 Rethinking the target: the double-edged sword of CSF1R blockade

The CSF1–CSF1R axis has been a major therapeutic target in fibrosis and cancer. However, the work of Vollmers et al (1). reveals a critical risk: indiscriminate blockade may deplete or incapacitate macrophages, withdrawing their homeostatic licensing influence and paradoxically triggering or worsening fibrosis. A temporal dimension is instructive here. Short-term inhibition may primarily suppress pro-inflammatory, monocyte-derived macrophages, providing benefit. Long-term blockade, however, risks depleting tissue-resident macrophages with homeostatic functions, exposing the host to a “licensing signal void” and a compensatory fibroblast rebound. In the TME, this risk has already manifested as CAF-induced compensatory recruitment of PMN-MDSCs (1, 39), a phenomenon that can be mitigated by the addition of a CXCR2 antagonist.

Extrapolating from oncology to fibrosis requires caution. The immune infiltrate and signaling network in fibrotic tissues differ substantially from those in tumors. In fibrosis, PMN-MDSCs are not the dominant compensatory population; instead, monocyte-derived pro-fibrotic macrophages (58)and activated fibroblasts form the core pathologic loop (59, 60). Consequently, combining CSF1R inhibition with a CXCR2 blocker may not yield the same benefit in fibrotic settings (61, 62). A more immediately translatable strategy in fibrosis may be to pair CSF1R modulation with agents that directly dampen fibroblast activation, such as YAP/TAZ inhibitors or anti-fibrotic cytokines (59, 63).

More broadly, a desirable strategy shifts from cell elimination to circuit rebalancing. Possible avenues include: (1) combination targeting, pairing a CSF1R inhibitor with agents that block compensatory fibroblast pathways; (2) phenotypic tuning, using approaches such as nanoparticle-delivered peroxisome proliferator-activated receptor gamma (PPAR-gamma), a nuclear receptor that promotes an anti-inflammatory macrophage phenotype, or signal transducer and activator of transcription 6 (STAT6) agonists, a transcription factor central to IL-4/IL-13-mediated alternative macrophage activation, to educate surviving macrophages toward a reparative, licensing-competent phenotype even in the face of CSF1R inhibition; and (3) signal fine-tuning, targeting downstream effectors such as YAP/TAZ rather than the upstream receptor, to achieve more precise regulation. Combining CSF1R modulation with agents that directly dampen fibroblast activation, such as YAP/TAZ inhibitors, is an attractive combinatorial concept. However, YAP/TAZ are broadly expressed transcriptional coactivators required for tissue regeneration, stem cell maintenance, and organ size control in epithelial, endothelial, and cardiac tissues. Systemic YAP/TAZ inhibition therefore carries a substantial risk of on-target toxicity in regenerating tissues. Achieving a therapeutic window will likely require targeted delivery strategies, for example, fibroblast-directed nanoparticle formulations, antibody-drug conjugates, or locally administered depot systems, to confine YAP/TAZ inhibition to the fibrotic stroma. The development of such engineered delivery systems remains an active area of pharmaceutical research and represents a prerequisite for translating this combinatorial approach into clinical practice. For the phenotypic tuning approach to succeed, macrophages within established fibrotic lesions must retain sufficient plasticity. Encouragingly, recent work shows that even chronically inflamed macrophages can be epigenetically reprogrammed when the driving stimulus is removed (64, 65), yet the dense, crosslinked extracellular matrix in fibrosis may limit the delivery of nanoparticle-based therapeutics, posing an additional hurdle that will need to be addressed by pharmaceutical engineering.

### 5.2 Key unanswered questions

1. The macrophage licensing signalome. What are the molecular messengers that keep fibroblasts quiescent? Systematic decoding—through conditional ligand libraries, receptor–ligand interaction screens, and multi-factorial *in vitro* reconstruction—is essential. As discussed above, candidate pathways include Wnt antagonists, BMPs, and the Gas6/Pros1–Axl axis, but direct *in vivo* evidence is lacking. In parallel, the contribution of active pro-fibrotic signals from Spp1^+^ or other emergent myeloid populations must be directly tested through lineage-specific neutralization.
2. Fibroblast subset division of labor and circuit topology. Do other fibroblast subsets, such as Pi16^+^ or Col15a1^+^ populations, manage distinct immunomodulatory tasks, and is there spatial or hierarchical organization among them?
3. Spatiotemporal circuit dynamics. How does circuit composition and spatial organization change across acute injury, chronic inflammation, and fibrosis? Spatiotemporally specific models that integrate the fibroblast response are needed.
4. Reversibility and therapeutic windows. Once circuit imbalance is established, is it relentlessly progressive, or does an intervenable tipping point exist? Determining whether functional licensing exhaustion is epigenetically fixed or therapeutically reversible is of paramount clinical importance.
5. Integration of microenvironmental signals. The skin and gut microbiomes, as well as mechanical forces such as cyclic respiratory stretch in the lung, can directly modulate pathways like YAP/TAZ. Understanding how these environmental inputs synergize with the cellular circuit will require a systems-level perspective.

## 6 Conclusions and perspective

The relationship between fibroblasts and macrophages extends far beyond a scaffold and its passive beneficiary. The discovery of *in vivo* reverse homeostatic licensing establishes the CSF1–CSF1R axis as the hub of a bidirectional information flow and constructs the framework of a “fibroblast–macrophage homeostatic circuit.” The balance of this circuit—the push of CSF1 and the pull of licensing signals—governs whether a tissue maintains quiet homeostasis, mounts a repair response, or slides into fibrosis. Critically, disruption can arise not only from numerical macrophage loss but also from a qualitative failure of licensing capacity, as we postulate occurs in chronic fibrotic diseases. From skin to lung, liver, and the tumor microenvironment, this circuit model provides a unified lens. More importantly, it forces a fundamental reevaluation of cell-depletion therapies. Indiscriminately targeting macrophages or fibroblasts may inadvertently unbalance the circuit. The next frontier is to move from a “cellular component” paradigm to a “cellular social network dynamics” paradigm, enabling the precise restoration of tissue equilibrium.
